# Comparative study of the oviduct of pre-laying and laying Egyptian balady ducks (*Anas boschas domesticus*) using morphometry, immunohistochemistry, scanning, and transmission electron microscopy: Abbreviated title: oviduct of pre-laying and laying Egyptian Balady ducks

**DOI:** 10.1186/s12917-025-04991-7

**Published:** 2025-10-03

**Authors:** Aya A. Abdelghany, Salah E. Elmorsy, Mesbah Abdelgawad, Galal A. Elsayed, Safwat Ebada, Ahmed M. Abdellatif

**Affiliations:** Department of Anatomy and Embryology, Faculty of Veterinary Medicine, Mansoura University, Mansoura, 35516 Egypt

**Keywords:** Anseriformes, Aves, Egg formation, Intraepithelial T cells, Oviduct, Ultrastructure

## Abstract

Egyptian balady duck (*Anas boschas domesticus*) is a breed of domestic duck native to Egypt of great economic importance. The present study investigated micromorphological, ultrastructural, and immunohistochemical aspects of the oviduct as well as serum estrogen and progesterone levels in pre-laying and laying Egyptian balady ducks. The oviduct comprised five segments: infundibulum, magnum, isthmus, uterus, and vagina. The mucosa was thrown into longitudinal folds throughout the oviduct, except for the vagina, where the folds appeared transverse. The folds were further split into secondary and tertiary folds. A significant age-associated increase in fold thickness was observed in the magnum and uterus. The surface epithelium appeared pseudostratified ciliated columnar, permeated by openings of the proprial glands. The secretory units of the proprial glands showed extensive branching in the magnum of laying ducks. Ultrastructurally, they revealed enhanced activity of Golgi apparatus and rough endoplasmic reticulum together with numerous spherical-shaped electron-dense granules in laying ducks compared to non-laying ones. Intracytoplasmic vacuoles were prominent in the surface epithelium, especially in the uterus, of pre-laying ducks and outnumbered those of laying ones. In contrast, the secretory cells of the surface epithelium appeared loaded with several electron-lucent granules concentrated toward their luminal side in laying ducks. CD3-immunoreactive T cells densely populated both the epithelium and lamina propria. A significantly higher presence of T cells was detected in the lamina propria than in the epithelium, regardless of duck age. Significantly higher serum estrogen and progesterone levels were detected in laying ducks compared to non-laying individuals, which may suggest a hormonal basis of the structural and ultrastructural differences between the two studied ages. The present study reported structural changes involving the oviduct associated with egg laying. This study improves the understanding of factors regulating avian oviduct health and productivity.

## Introduction

Poultry eggs represent a common source of human nutrition. They are enriched with essential amino acids, polyunsaturated fatty acids, vitamins, and minerals [1]. The avian oviduct is the biological assembly line of eggs in which successive layers are added to the developing egg. Anatomically, the avian oviduct extends from the ovary to the cloaca and is formed from five chief segments, namely the infundibulum, magnum, isthmus, uterus (shell gland), and vagina [2]. The infundibulum is the initial part of the oviduct. It is responsible for reception of ovum, through action of its fimbriae, and formation of the perivitelline membrane and chalaza around it. The magnum is the longest and most tortuous part of the duct and is concerned with the production of egg albumin. The isthmus is a narrow part that precedes the uterus and is responsible for the formation of the shell membranes. The uterus is a dilated part of the duct and is involved in the deposition of the eggshell [3]. The vagina is the terminal part of the oviduct in which the formed egg relays briefly before its release into the cloaca and subsequently to the exterior [4].

Histologically, the oviduct wall is formed throughout its length of four distinct concentric layers: mucosa, submucosa, muscularis, and serosa. The mucosa is arranged into mucosal folds that assist in egg passage inside the duct and consists of an epithelium of variable height and a glandular lamina propria. The epithelium ranges from simple flattened to pseudostratified columnar. The columnar cells comprise ciliated, non-ciliated secretory, and basal cells. The lamina propria contains tubular glands throughout its length. The tunica muscularis contains circular and longitudinal muscle layers; their peristaltic contractions also assist in egg transportation [5].

The processes of egg formation and laying are primarily dependent on the health and integrity of the oviduct wall. Indeed, altered egg production is a common feature of several disease conditions of both reproductive and non-reproductive origins [6]. Therefore, identifying the basic structure of various oviduct segments is crucial for proper interpretation of these conditions. Microanatomical features of the oviduct in domestic birds have been reported in chicken [7], turkey [8], pigeon [9], and quail [10]. Data on the gross anatomy of the Egyptian balady ducks were referred to by a previous report [11]. However, in-depth analyses of micromorphological and ultrastructural features of the oviduct in Egyptian balady ducks, especially those triggered by egg laying, are lacking.

Ducks are a popular human food in many developing countries of the world. Particularly, in Egypt there is a gap between annual production, ~ 10 million ducks, and the actual needs, ~ 35 million [12]. Thus, a search for factors that improve duck productivity is ongoing. Egyptian balady duck (*Anas boschas domesticus*) is a main duck breed native to Egypt characterized by its palatable meat and adaptation to a wide range of atmospheric conditions. Compared to chickens, balady ducks exhibit a higher degree of natural resistance to certain diseases due to inherent genetic factors [13]. Moreover, it mandates fewer maintenance costs compared to other duck breeds, making them a good candidate for producing high-quality meat at low economic risk. The shift from pre-laying to laying state is known to involve biological changes in the secretory and immunological milieu of the oviduct. Mucosal T cells regulate local actions against infectious agents via potent cytokine- and chemokine-mediated immune responses [14, 15]. Characterizing the distribution pattern of these cells in avian species under basal conditions will help in interpreting their changes during various inflammatory conditions. In view of this, the present study aimed to compare the gross anatomy, morphometry, ultrastructure, and T cell distribution pattern of the oviduct of juvenile and in-lay Egyptian balady ducks.

## Materials and methods

### Birds and ethical statement

The material of the present study included 42 oviducts of apparently healthy Egyptian balady ducks (*Anas boschas domesticus*) (21 were 5-month-old (pre-laying); 21 were 8-month-old (laying)). These birds were purchased from local breeders in Dakahlyia governorate, Egypt. This study was permitted by the Animal Care and Use Committee of Mansoura University (MU-ACUC) (VM.PhD.23.11.31). The study agreed with the ARRIVE guidelines and complied with the National Institutes of Health Guide for the Use of Animals in Research (NIH Publications No. 8023, revised 1978).

### Gross anatomy

Birds were humanely euthanized via cervical dislocation and bled instantly [16]. Immediately after death confirmation, the oviduct was located, photographed, and extracted. Next, oviduct dimensions were estimated using a digital Vernier caliper (VINCA DCLA-0605, Clockwise Tools Inc., Valencia, CA, USA).

### Histological analysis

Different parts of the Egyptian duck oviduct (infundibulum, magnum, isthmus, uterus, and vagina) of five birds from both ages were transversely cut into 15 mm long specimens and placed in labeled plastic cassettes. Tissue specimens were then fixed in 10% neutral buffered formalin for one week before being processed for paraffin embedding using standard histological techniques [17]. The paraffin blocks were sectioned using a rotary microtome to obtain 4 μm thick sections. The sections were stained with Harris’ hematoxylin and eosin (H&E) stain for demonstration of general histological structures and periodic acid-Schiff (PAS) stain for neutral mucopolysaccharides [18].

### Morphometric study

The morphometric analysis in the present study focused on various oviduct segments of both pre-laying and laying ducks. Parameters measured included the width and length of mucosal folds, thickness of the proprial glands, and height of the surface epithelium. These measurements were obtained using the measuring tool of ImageJ2 software (ver. 2.14/1.54f) [19]. Twenty-five transversely cut sections collected 500 μm apart were analyzed per each oviduct segment.

### Scanning electron microscopy

Scanning electron microscopy was used to study the surface ultrastructure of the five oviduct segments of both pre-laying and laying ducks as previously described [20, 21]. Briefly, tissue specimens were trimmed, rinsed in phosphate buffer (pH 7.4), and fixed in a solution containing 2.5% paraformaldehyde and 2.5% glutaraldehyde (pH 7.3). Specimens were subsequently post-fixed in 1% osmium tetroxide, dehydrated through a graded ethanol series, and then dried using a critical point drying chamber. Dried specimens were coated with gold particles and examined using a JSM-6510 LV scanning electron microscope (JEOL Ltd., Tokyo, Japan).

### Transmission electron microscopy

Transmission electron microscopy was employed to study the cellular details of the five oviduct segments of both pre-laying and laying ducks as previously described [22]. Briefly, specimens were cut into smaller pieces (1 mm x 1 mm) and fixed in a solution containing 2.5% glutaraldehyde and 2% paraformaldehyde in phosphate buffer pH (7.4). The specimens were then re-fixed in 1% osmium tetroxide for 1–2 h. Specimens were immersed in ascending grades of ethanol for dehydration, acetone Epon mixtures for infiltration, and Epon for embedding. Ultrathin sections. (70 nm) were cut by means of an ultramicrotome and incubated with uranyl acetate and lead citrate for staining. The stained sections were finally analyzed and photographed using a JEM − 2,100 transmission electron microscope (JEOL Ltd., Tokyo, Japan).l

### CD3 immunohistochemistry

Immunohistochemical staining was performed using the DAB method on dewaxed paraffin sections as previously described [23, 24]. Briefly, dewaxed sections were washed thrice in PBS and microwaved in citrate buffer (pH = 6) for 20 min to revive the antigenic epitopes. Next, the sections were covered with 5% bovine serum albumin (BSA) for 1 h to block nonspecific binding sites before addition of the primary antibody, goat polyclonal anti-CD3 (sc-1127, 1:1000, Santa Cruz Biotechnology Inc., Dallas, TX, USA). Following a proper wash in PBS, biotinylated secondary antibody (Jackson ImmunoResearch, West Grove, PA, USA) was applied for 1 h to ensure binding to the primary antibody. A 0.3% H_2_O_2_ solution flooded the sections for 20 min to quench their endogenous peroxidase activity. Reaction intensity was further enhanced using VECTASTAIN Elite kit (PK-6100, Vector Laboratories, Burlingame, CA, USA) per manufacturer’s instructions. Freshly prepared diaminobenzidine solution (SK-4103, Vector Laboratories) was used to visualize the reaction. All sections were finally counterstained in hematoxylin and examined under a light microscope. Eight microscopic images (40x) were captured from each specimen. CD3 immunoreactive cells were analyzed using the point counting tool of ImageJ.

### Analysis of serum Estrogen and progesterone levels

Blood was withdrawn from the brachial vein of 12 unanesthetized ducks (six per age). To minimize fluctuations in hormone levels, blood collection was performed in the morning for all birds and 2 h before ovulation in case of laying ducks. The time of ovulation was roughly estimated based on the timing of egg deposition on the previous day. Blood samples were centrifuged at 3500 rpm for 10 min, and the serum was decanted. Levels of estradiol, the most active form of estrogen (06656021190, Roche Diagnostics, Mannheim, Germany), and progesterone (07092539190, Roche Diagnostics) were estimated using commercial electrochemiluminescence immunoassays according to the manufacturer’s instructions.

### Statistical analysis

Data were analyzed in GraphPad Prism 8 (GraphPad Software, La Jolla, CA, USA). Differences in parameters of each oviduct segment and serum reproductive hormone levels of pre-laying and laying ducks were determined using the student’s t-test. Results were shown as the mean ± SD. *P* < 0.05 was used to express statistical significance.

## Results

### Gross anatomy

**Fig. 1:**
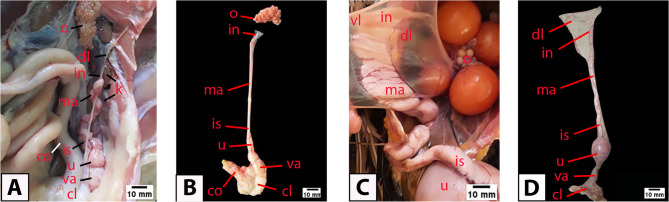
Gross appearance of the oviduct in pre-laying (**A** and **B**) and laying (**C **and **D**) Egyptian balady duck. Note the progressive increase in the oviduct diameter with age. cl, cloaca; co, colon; dl, dorsal oviductal ligament; in, infundibulum; is, isthmus; k, kidney; ma, magnum; o, ovary; u, uterus; va, vagina; vl, ventral oviductal ligament

The oviduct of the Egyptian balady duck appeared as a single tubular structure of variable diameter passing along the dorsal aspect of the celomic cavity. It was related to the left kidney dorsally, colon medially, ovary cranially, and cloaca caudally (Fig. 1A). The oviduct consisted of five segments: infundibulum, magnum, isthmus, uterus, and vagina (Fig. 1B). The infundibulum consisted of a cranial funnel-shaped transparent part and a caudal thicker tubular part. The magnum was the longest and most convoluted part of the duct. The isthmus appeared short and constricted. The uterus appeared significantly larger in laying ducks compared to pre-laying ones (Fig. 1B, C, D). The vagina was the terminal segment of the oviduct and appeared connected to the cloaca (Fig. 1B, D). The oviduct is fixed to the dorsal abdominal wall by the dorsal oviductal ligament and to the ventral abdominal wall by the ventral oviductal ligament (Fig. 1A, C).

**Fig. 2:**
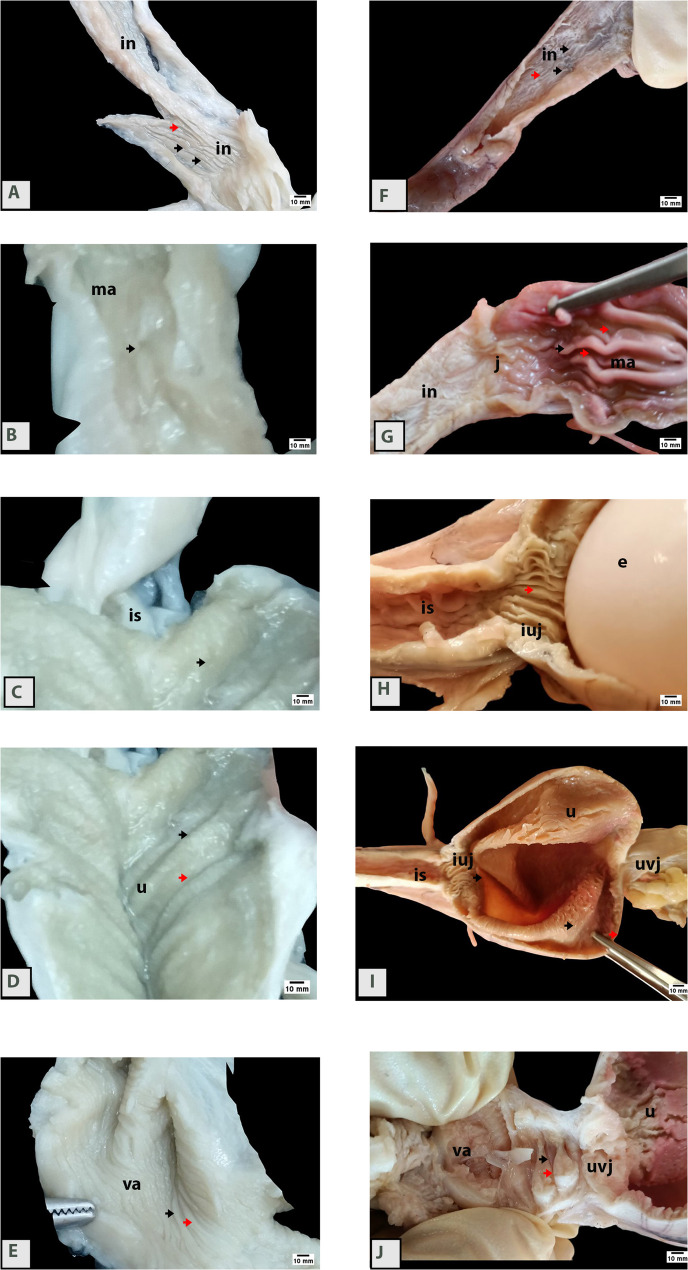
Gross appearance of parts of the oviduct in pre-laying (**A, B, C, D, E**) and laying (**F, G, H, I, J**) Egyptian balady duck. Note oviductal mucosa demarcated into mucosal folds (black arrows) separated from each other’s by grooves (red arrows). Such demarcation appears more apparent in laying ducks compared to the pre-laying ones. in, infundibulum; is, isthmus; iuj, isthmo-uterine junction; ma, magnum; u, uterus containing an egg (e) in H; uvj, uterovaginal junction; va, vagina

The oviductal mucosa of the Egyptian balady duck was distinctly partitioned into mucosal folds, each separated by well-defined grooves (Fig. 2A, B, C, D, E). The folds appeared longitudinally oriented in the infundibulum, magnum, isthmus, and uterus but transverse in the vagina. The demarcation of the folds appears less apparent in prelaying ducks compared to the laying ones (Fig. 2F, G, H, I, J). Mucosal folds in the magnum and uterine regions were significantly taller in laying ducks than in pre-laying individuals (Fig. 2G, H, I).

### Histology and surface ultrastructure

**Fig. 3:**
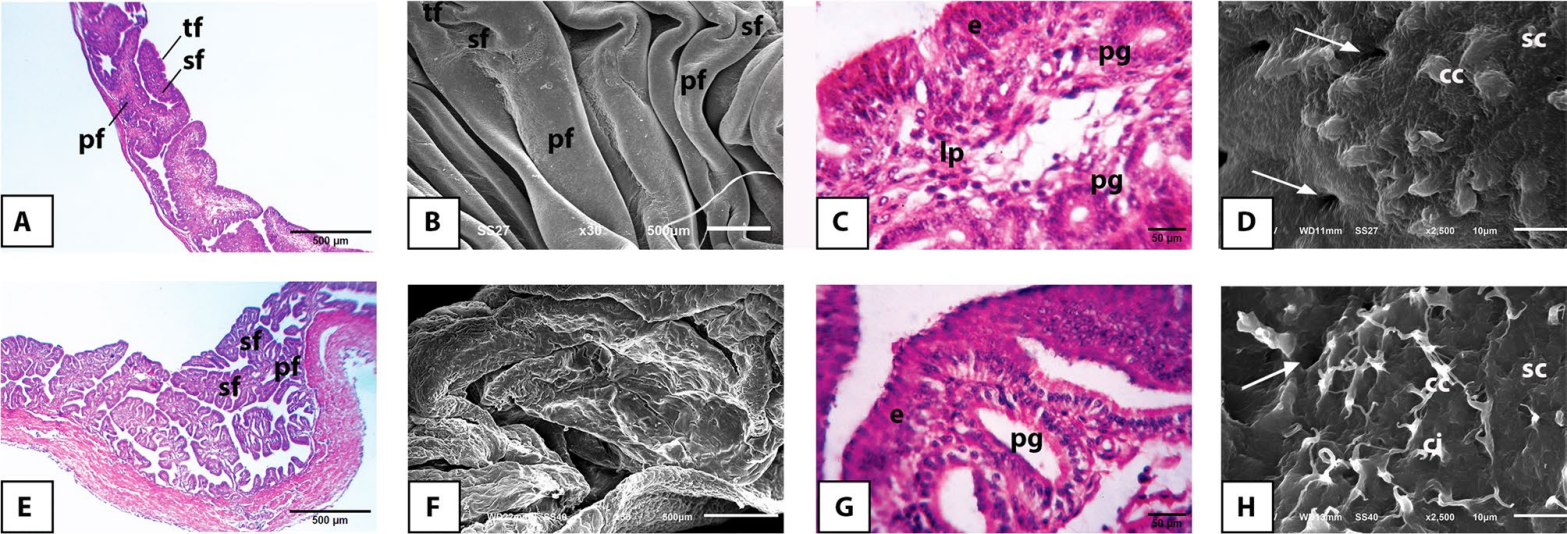
Light and scanning electron microscopic appearance of the infundibulum of the pre-laying (**A-D**) and laying (**E-H**) Egyptian balady duck. **A** Light micrograph (LM) showing the primary (pf), secondary (sf), and tertiary (tf) mucosal folds. H&E stain. **B** Scanning electron micrograph (SEM) showing the different order of mucosal folds. **C** LM showing the pseudostratified epithelium (e) covering a mucosal fold and the proprial glands (pg) within its lamina propria (lp). H&E stain. **D** SEM of the infundibular epithelium showing the ciliated (cc) and secretory (sc) cells. Glandular openings are denoted by arrows. **E** LM showing the primary (pf) and secondary (sf) folds. H&E stain. **F** SEM showing the composite arrangement of the mucosal folds. **G** LM showing the proprial glands (pg) approaching the epithelium (e). H&E stain. **H** SEM showing the ciliated cells (cc) bearing prominent cilia (ci). Sc, secretory cells. Arrow refers to a glandular opening

The mucosa of the infundibulum of the Egyptian balady duck was arranged into mucosal folds of primary, secondary, and tertiary order and lined with pseudostratified columnar ciliated epithelium overlying a compact connective tissue core. The latter enclosed the tubular gland in both prelaying (Fig. 3A, B, C) and laying (Fig. 3E, F, G) ducks. Ultrastructurally, the surface epithelium revealed openings of the proprial glands and was clearly distinguished into ciliated and non-ciliated cells. The former revealed a distinctly developed cilia in the laying ducks (Fig. 3D, H).

**Fig. 4:**
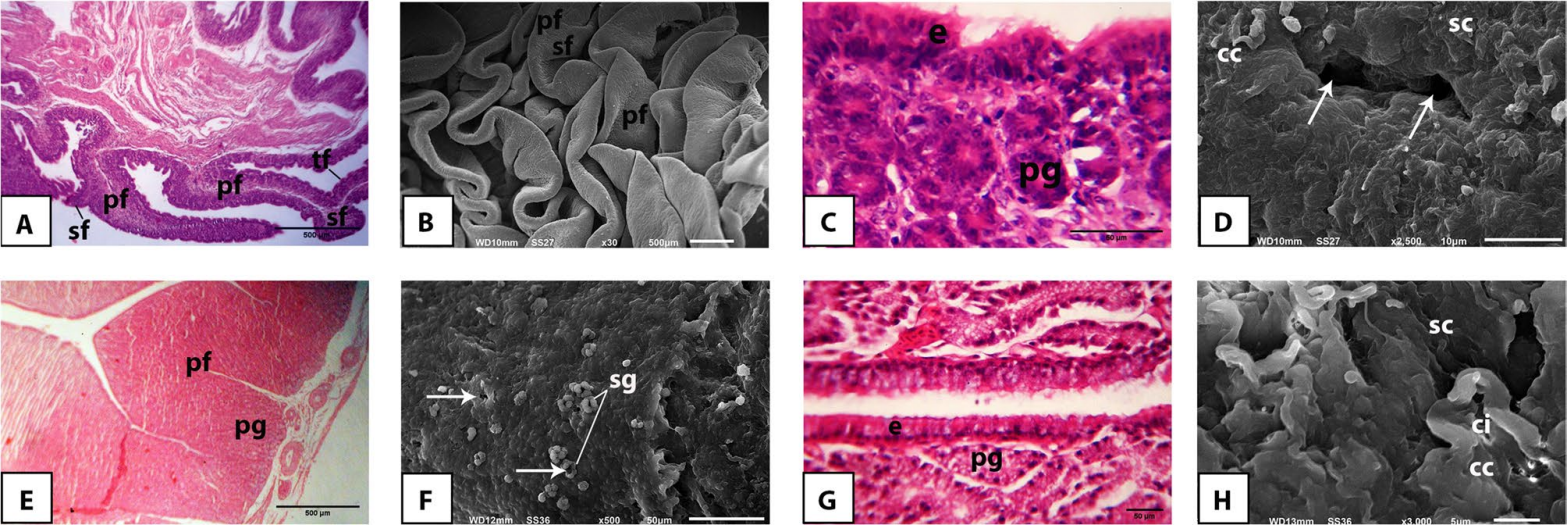
Light and scanning electron microscopic appearance of the magnum of the pre-laying (**A-D**) and laying (**E-H**) Egyptian balady duck. **A** Light micrograph (LM) showing long and slim mucosal folds of primary (pf), secondary (sf), and tertiary (tf) order. H&E stain. **B** Scanning electron micrograph (SEM) showing the different order of mucosal folds. **C** LM showing the epithelium (e) and proprial glands (pg). H&E stain. **D**) SEM showing the ciliated (cc) and secretory (sc) cells intervened by openings of the proprial glands (arrows). **E** LM showing a thickened primary fold (pf). H&E stain. **F** SEM showing secretory granules (sg) in vicinity of the glandular openings (arrows). **G** LM showing the active and vacuolated cytoplasm of the proprial glands (pg); e, epithelium. H&E stain. **H** SEM showing the ciliated cells (cc) bearing long cilia (ci); sc, secretory cells

The mucosa of the magnum of the Egyptian balady duck revealed a similar pattern of arrangement of mucosal folds and appearance of lining epithelium to that of the infundibulum in both prelaying (Fig. 4A, B, C, D) and laying (Fig. 4E, F, G, H) ducks. Increased fold thickness, vacuolated cytoplasm of the surface and glandular epithelia, high number of densely packed and branched tubular glands, and abundant secretory granules in the vicinity of gland openings on the surface epithelium were the most prominent features of the magnum in laying ducks (Fig. 4E, F, G).

**Fig. 5:**
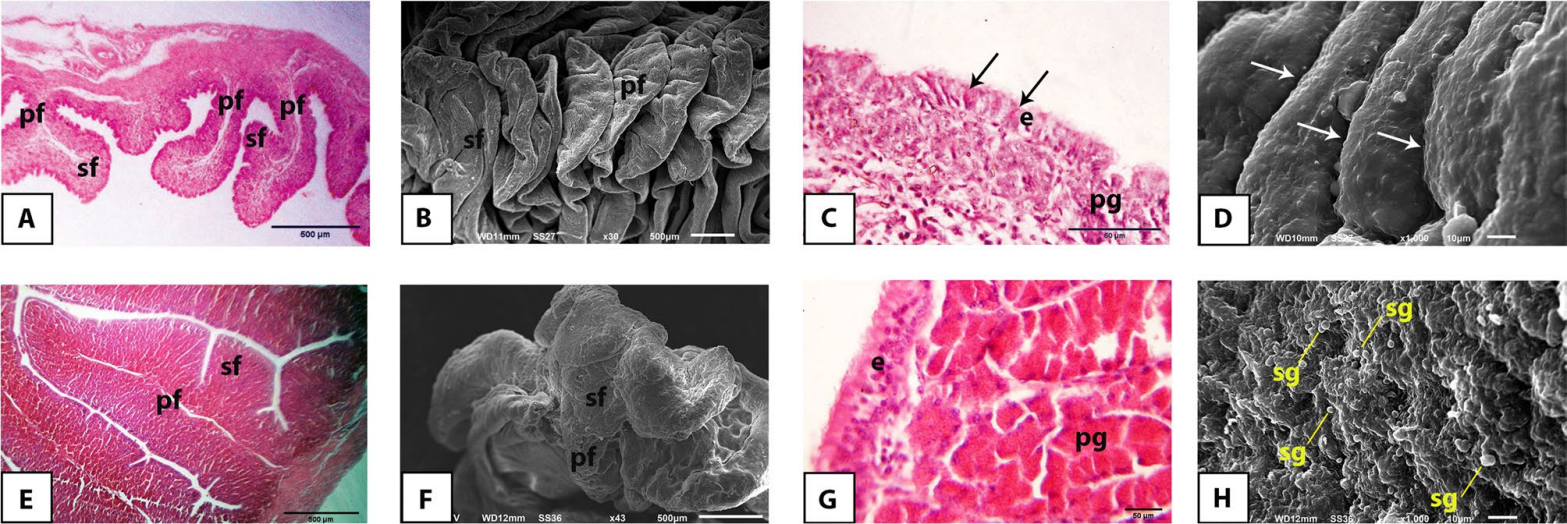
Light and scanning electron microscopic appearance of the isthmus of the pre-laying (**A-D**) and laying (**E-H**) Egyptian balady duck. **A** Light micrograph (LM) showing primary (pf) and secondary (sf) mucosal folds. H&E stain. **B** Scanning electron micrograph (SEM) showing leaf-like primary (pf) and secondary (sf) folds. **C** LM showing PAS-positive staining of the secretory cells (arrows); e, epithelium; pg, proprial glands. PAS stain. **D** SEM showing deep furrows (arrows) separating the mucosal folds. **E** LM showing thick primary (pf) and secondary (sf) mucosal folds. H&E stain. **F** SEM showing the appearance of the folds. **G** LM showing the acidophilic cytoplasm of the proprial glands (pg); e, epithelium. H&E stain. **H** SEM showing multiple secretory granules (sg) covering the surface epithelium

The mucosa of the isthmus of the Egyptian balady duck was arranged into primary and secondary mucosal folds in both prelaying (Fig. 5A, B) and laying (Fig. 5E, F) ducks. Compared to prelaying ducks, the glandular epithelium of the isthmus of laying ducks appeared more eosinophilic and covered by secretory granules of higher abundance in laying ducks (Fig. 5C, D, G, H).

**Fig. 6:**
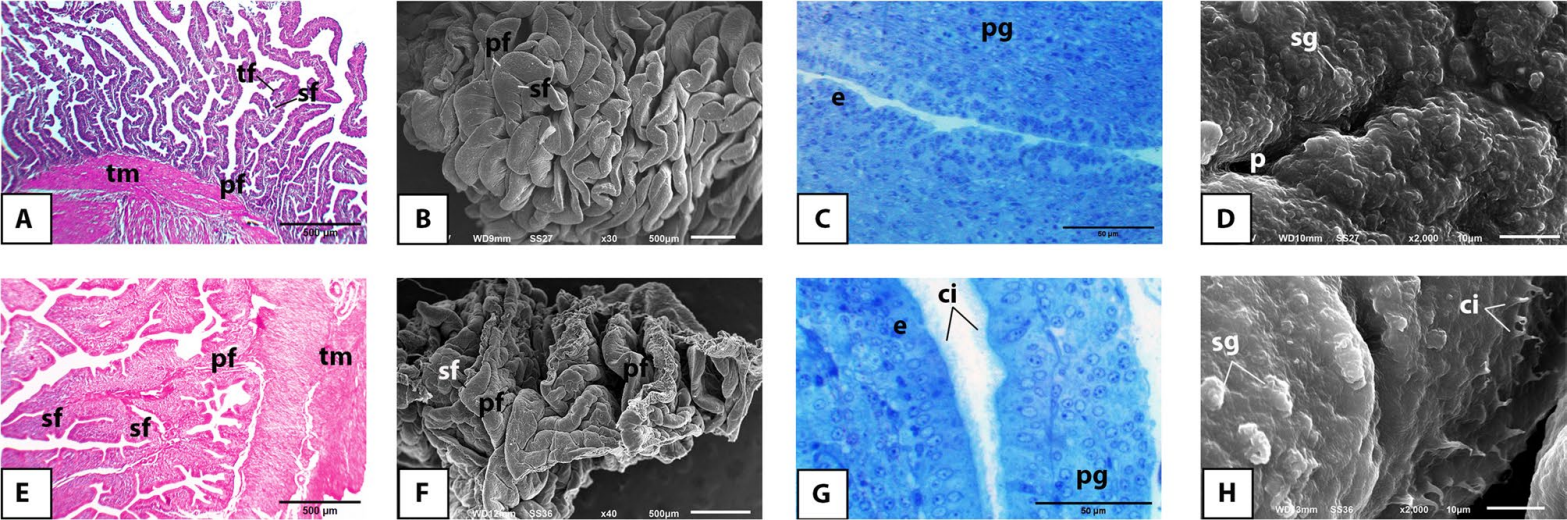
Light and scanning electron microscopic appearance of the uterus of the pre-laying (**A-D**) and laying (**E-H**) Egyptian balady duck. **A** Light micrograph (LM) showing primary (pf), secondary (sf) and tertiary (tf) mucosal folds. tm, tunica muscularis. H&E stain. **B** Scanning electron micrograph (SEM) showing the appearance of the folds. **C** LM showing the epithelium (e) and proprial glands (pg). Toluidine blue stain. **D** SEM demonstrating presence of secretory granule (sg) close to a proprial gland opening (p). **E** LM showing branched and convoluted primary (pf) and (sf) folds. **F** SEM showing the appearance of the folds. **G** LM showing the epithelium (e) with prominent cilia (ci), and the proprial glands (pg). Toluidine blue stain. **H** SEM showing well-developed cilia (ci) and numerous secretory granules (sg) of the laying duck epithelium

The mucosa of the uterus of the Egyptian balady duck was arranged into mucosal folds of primary, secondary, and tertiary orders in both prelaying (Fig. 6A, B) and laying (Fig. 6E, F) ducks. The surface epithelium revealed more prominent cilia in the laying ducks compared to juvenile ones (Fig. 6C, D, G, H).

**Fig. 7:**
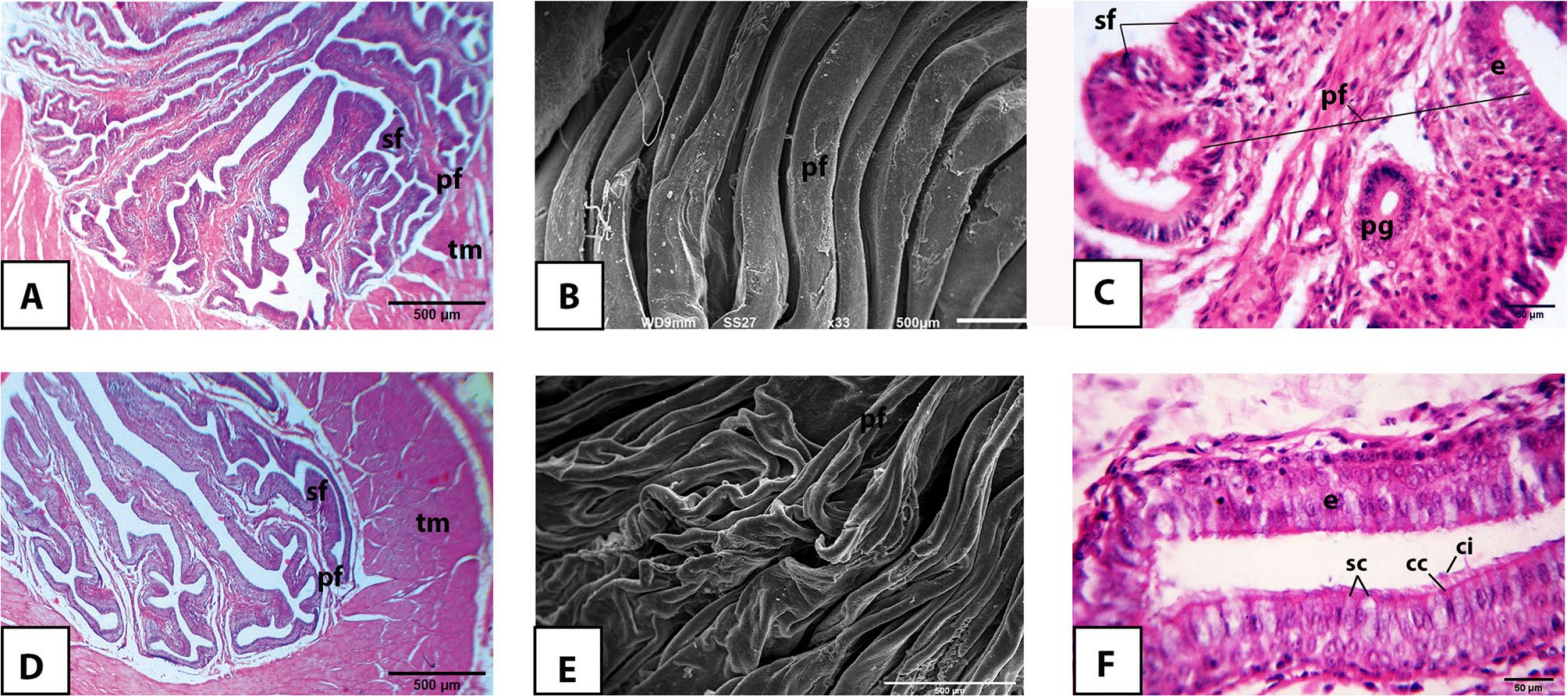
Light and scanning electron microscopic appearance of the vagina of the pre-laying (**A-C**) and laying (**D-F**) Egyptian balady duck. **A** Light micrograph (LM) showing primary (pf) and secondary (sf) mucosal folds; tm, tunica muscularis. H&E stain. **B** Scanning electron micrograph (SEM) showing the appearance of the mucosal folds. **C** LM showing a primary fold (pf) giving rise to two secondary folds (sf); e, epithelium; pg, proprial glands. H&E stain. **D** LM showing primary (pf) and secondary (sf) mucosal folds; tm, tunica muscularis. H&E stain. **E** SEM showing the appearance of the mucosal folds. **F** LM showing the vaginal epithelium (e) clearly formed of ciliated (cc) and secretory cells (sc); ci, cilia

The vaginal mucosa of the Egyptian balady duck was made up of long, narrow, branched, and leaf-like folds of primary and secondary orders in both prelaying (Fig. 7A, B) and laying (Fig. 7D, E) ducks. The surface epithelium appeared more corrugated in prelaying ducks (Fig. 7A, C) than in laying ones (Fig. 7D, F).

### Morphometry

**Fig. 8:**
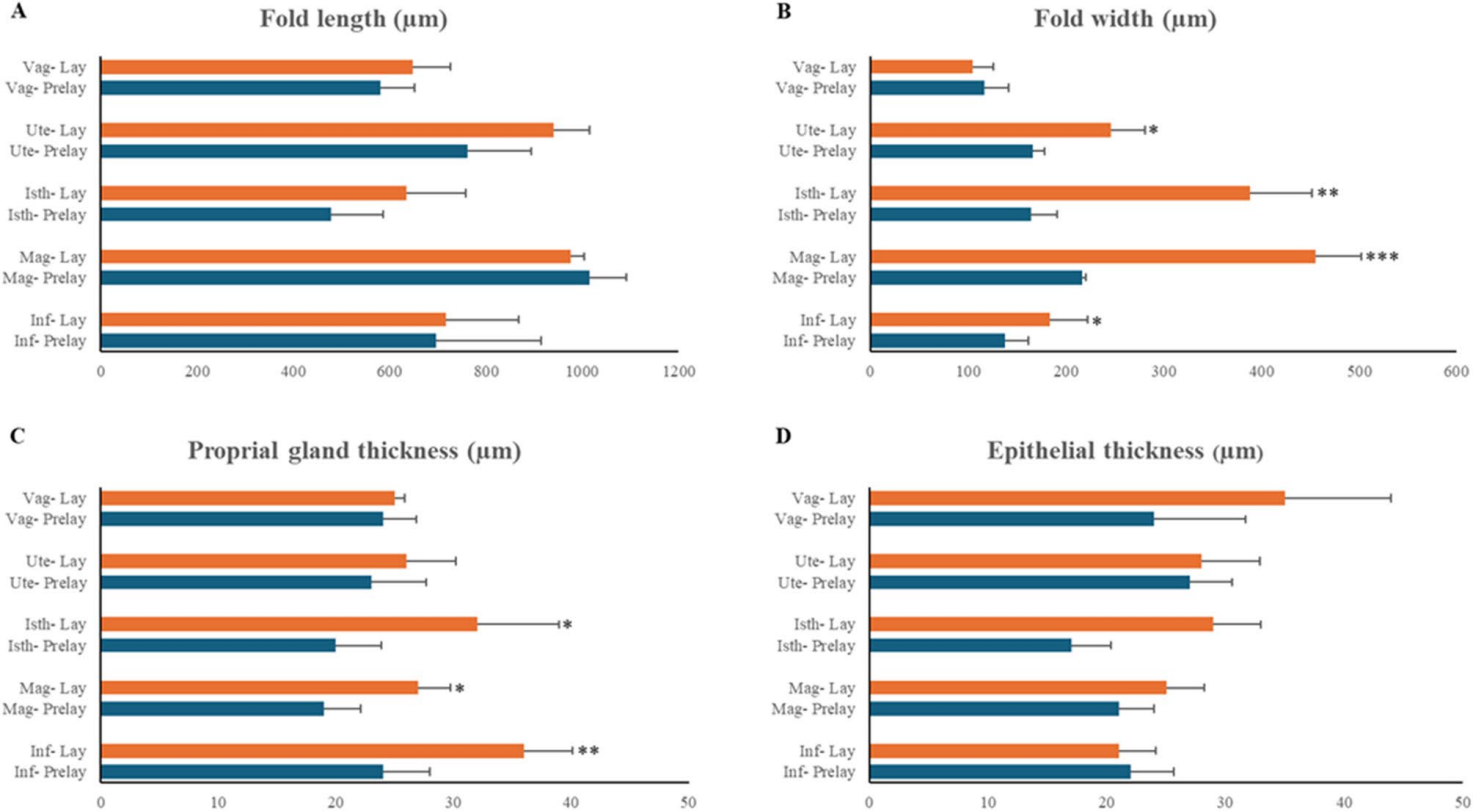
Bar charts of morphometric measurements of different oviduct segments of the pre-laying (Prelay) and laying (Lay) Egyptian balady duck. **A** Mucosal fold length. **B** Mucosal fold width. **C** Proprial gland thickness. **D** Surface epithelium thickness. Inf, infundibulum; Mag, magnum; Isth, isthmus; Ute, uterus; Vag, vagina. Data are presented as mean ± SD. *, *P* < 0.05. **, *P* < 0.01. ***, *P* < 0.001

Morphometric data on mucosal fold length, mucosal fold width, proprial gland thickness, and surface epithelium height in the oviduct of prelaying and laying Egyptian balady ducks are shown in Table 1. The length of mucosal folds seemed to be not affected by egg laying, and its values appeared comparable between prelaying and laying ducks (Fig. 8A). On the other hand, the width of the mucosal folds was significantly greater in the laying ducks, especially the magnum, followed by the isthmus, uterus, and infundibulum (Fig. 8B). The increase in the fold thickness is coupled with an increase in the proprial gland thickness in the infundibulum, magnum, and isthmus (Fig. 8C). The height of the surface epithelium was minimally changed between the two studied groups (Fig. 8D).

**Table 1:** Morphometric measurements of oviduct in prelaying and laying Egyptian balady ducks

Oviduct segment	Fold length	*P* value	Fold width	*P* value	PG thickness	*P* value	Epithelial thickness	*P* value
Inf- Prelay	696 ± 218	0.84	138 ± 23	**0.016**	24 ± 3.9	**0.009**	22 ± 3.6	0.67
Inf- Lay	717 ± 151		184 ± 38		36 ± 4.1		21 ± 3.1	
Mag- Prelay	1015 ± 91	0.89	217 ± 4	**0.0003**	19 ± 3.1	**0.012**	21 ± 3.0	0.58
Mag- Lay	976 ± 38		455 ± 47		27 ± 2.7		25 ± 3.2	
Isth- Prelay	478 ± 108	0.45	164 ± 27	**0.006**	20 ± 3.9	**0.035**	17 ± 3.4	0.17
Isth- Lay	635 ± 124		388 ± 64		32 ± 6.9		29 ± 4.0	
Ute- Prelay	762 ± 131	0.26	166 ± 12	**0.022**	23 ± 4.6	0.42	27 ± 3.6	0.76
Ute- Lay	940 ± 75		246 ± 35		26 ± 4.2		28 ± 4.9	
Vag- Prelay	580 ± 73	0.77	117 ± 24	0.067	24 ± 2.8	0.65	24 ± 7.7	0.25
Vag- Lay	647 ± 79		105 ± 21		25 ± 0.9		35 ± 9.0	

All listed measurements are in µm. Parameters revealing statistical differences (*P* < 0.05) are shown in bold. *Prelay* prelaying, *Lay* laying, *PG* proprial gland, *Inf* infundibulum, *Mag* magnum, *Isth* isthmus, *Ute* uterus, *Vag* vagina

### Transmission electron microscopy

**Fig. 9:**
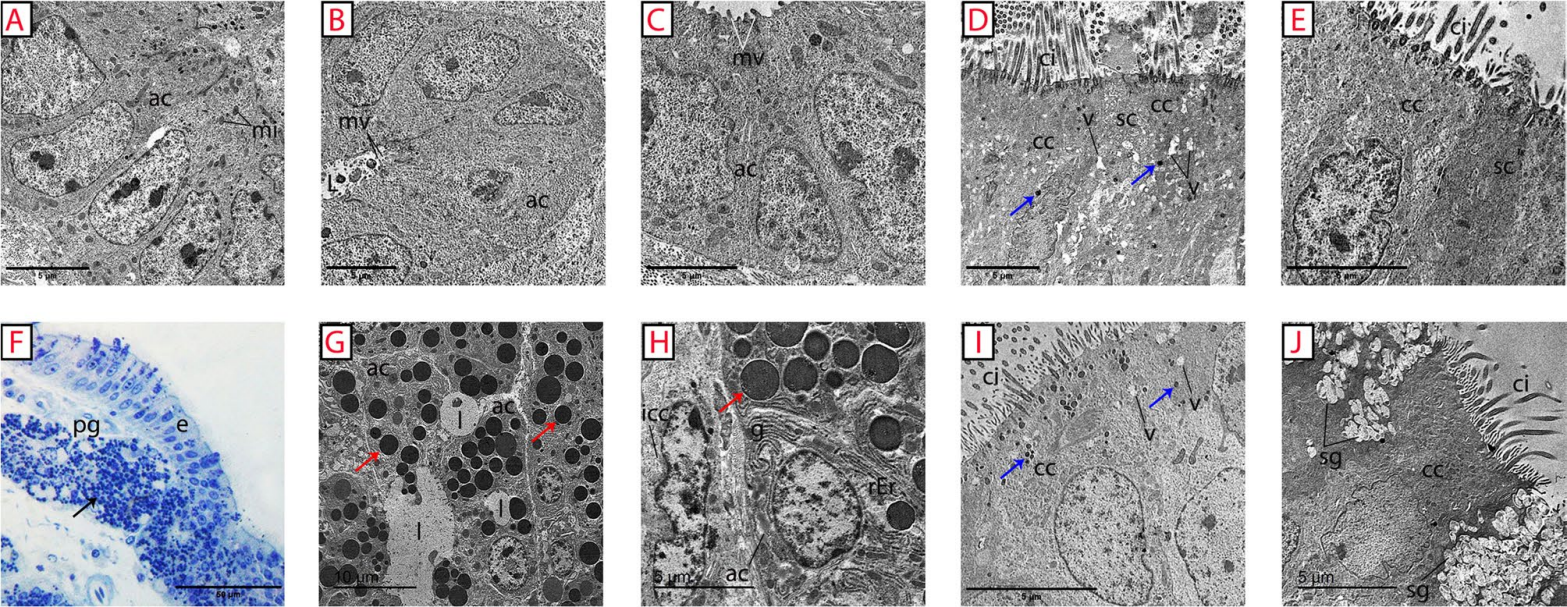
Main ultrastructural features of the mucosa of different oviduct segments of the pre-laying (**A-E**) and laying (**F-J**) Egyptian balady duck. **A**,** F** infundibulum; glandular part. **B**,** G** Magnum; glandular part. **C**,** H** Isthmus; glandular part. **D**,** I** Uterus; epithelium. **E**,** J** Vagina; epithelium. Note the remarkably higher activity of the proprial glands in laying ducks, indicated by dark cytoplasmic granules in toluidine blue-stained semithin section of the infundibulum in F (black arrow) and electron dense granules (red arrows) in transmission electron micrographs of the magnum (G) and isthmus (H), compared to pre-laying ones. Secretory granules of the surface epithelial cells are referred to by blue arrows. ac, secretory cells of proprial glands; cc, ciliated cells; ci, cilia; e, epithelium; g, Golgi apparatus; icc, interstitial Cajal-like cells; l, glandular lumen; mi, mitochondria; mv, microvilli; pg, proprial glands; rER, rough endoplasmic reticulum; sc, secretory cells; sg, secretory granules

Transmission electron microscopy was employed to examine the ultrastructural features of the mucosa of different oviduct segments of the pre-laying (Fig. 9A, B, C, D, E) and laying (Fig. 9F, G, H, I, J) Egyptian balady duck. The cells forming the secretory units of the proprial glands in pre-laying ducks contained oval-shaped nuclei and abundant mitochondria and carried microvilli along their free borders (Fig. 9A, B, C). Intracytoplasmic secretory granules were seldom detected. Conversely, numerous spherical-shaped electron-dense granules were observed within the secretory cells of proprial glands in laying ducks (Fig. 9G, H). The existence of these granules within the latter was accompanied by the presence of a well-developed Golgi complex and rough endoplasmic reticulum working continuously for their production and packaging (Fig. 9H). Interstitial Cajal-like cells, or telocytes, with their characteristic spindle-shaped outlines and single elongated nucleus surrounded by several mitochondria, were occasionally encountered in the vicinity of the secretory units of the proprial glands (Fig. 9H).

Membranless intracytoplasmic vacuoles or vacuoloides were prominent in the surface epithelium, especially in the uterus, of pre-laying ducks and outnumbered those of laying ones (Fig. 9D, I). On the other hand, the surface epithelial cells appeared loaded with numerous secretory granules that appeared concentrated toward their luminal aspect in laying ducks. The latter were prominent within the vaginal mucosa (Fig. 9J).

### CD3 immunoreactivity

**Fig. 10:**
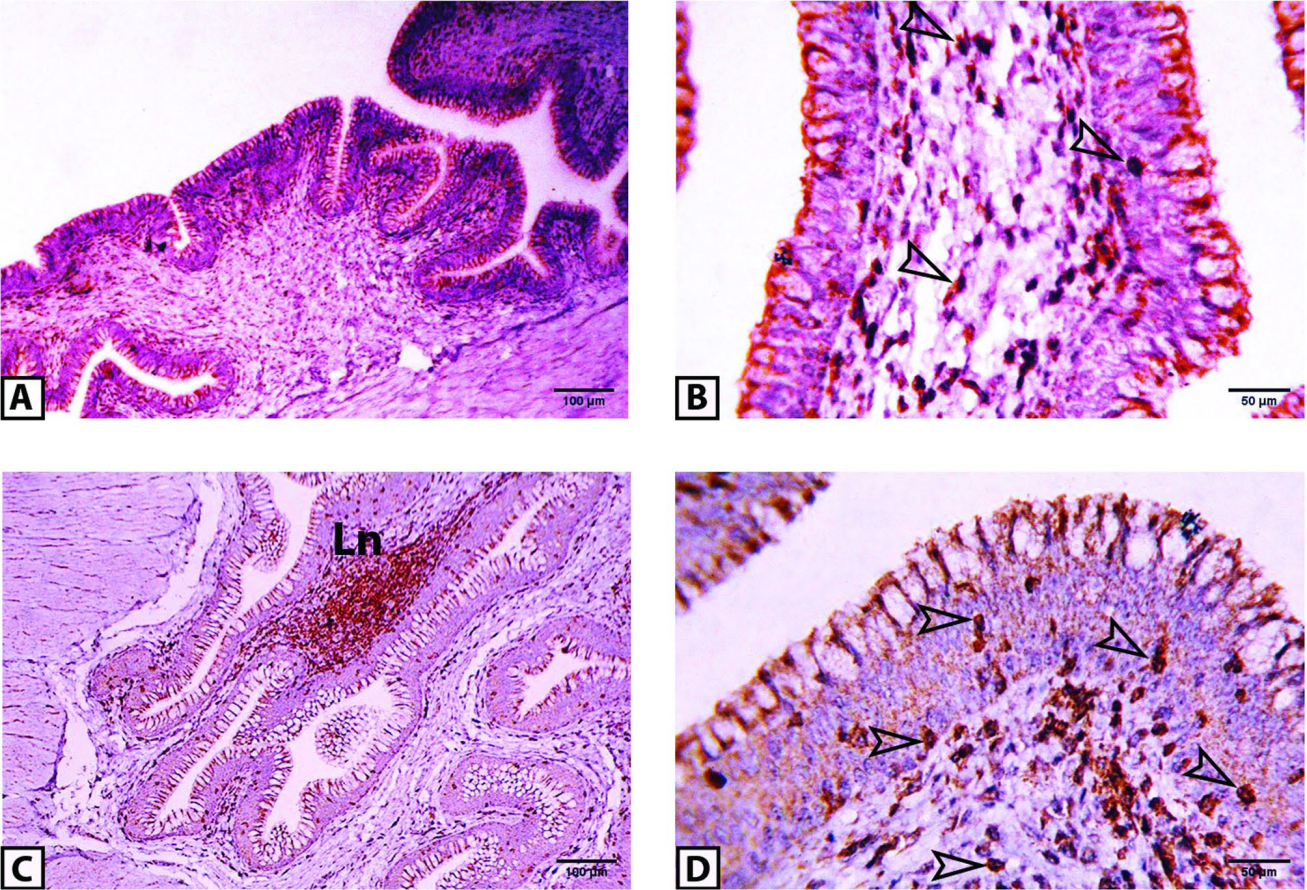
Representative light micrographs for the distribution of CD3 immunoreactive T lymphocytes within the vaginal mucosa of the pre-laying (**A, B**) and laying (**C, D**) Egyptian balady duck. CD3 immunoreactive cells (empty arrowheads) are seen within both the epithelium and lamina propria. Ln, lymphoid nodule

**Fig. 11:**
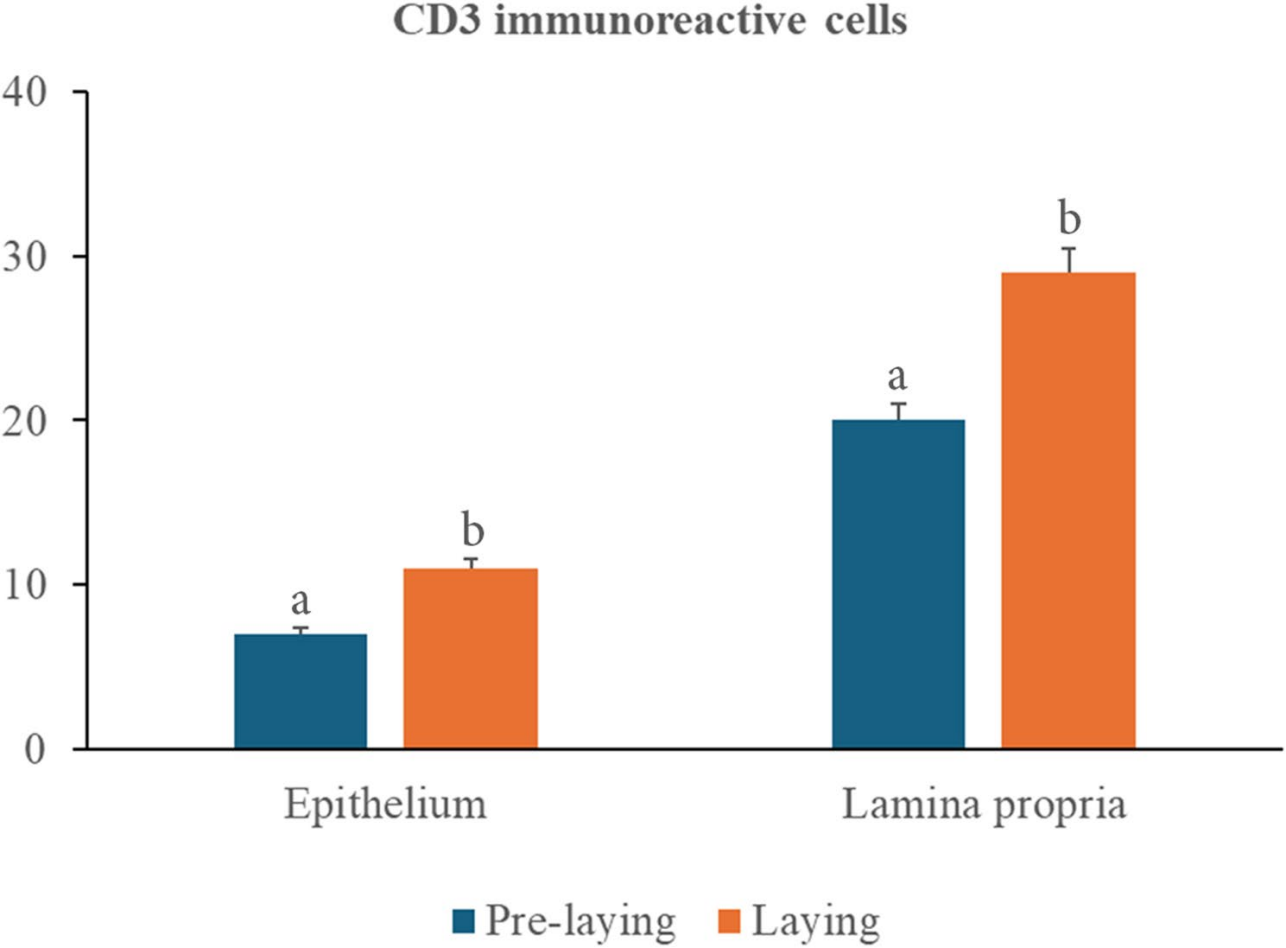
Quantitative comparison of the number of CD3 immunoreactive T lymphocytes within the vaginal mucosa of the pre-laying (blue) and laying (orange) Egyptian balady duck. Different superscripts indicate statistical significance, *p* < 0.05

A dense population of CD3-immunoreactive T cells was observed in both the epithelial layer and lamina propria of the vaginal wall (Fig. 10). Quantitative assessment demonstrated a significantly greater abundance of these cells in the lamina propria (20 ± 2.7 in prelaying ducks vs. 29 ± 4.2 in laying ducks, *P* < 0.01) than in the epithelium (7 ± 0.9 in prelaying ducks vs. 11 ± 1.4 in laying ducks, *P* < 0.01), regardless of the ducks’ age (Fig. 11).

### Serum reproductive hormone changes

Biochemical evaluation of serum primary reproductive hormones revealed differential expression of their concentrations between prelaying and laying Egyptian balady ducks. Significantly higher estrogen (*P* < 0.001) and progesterone (*P* < 0.0001) concentrations were evident in laying ducks (Table 2).

**Table 2:** Serum estrogen and progesterone levels in prelaying and laying Egyptian balady ducks

Hormone	Prelaying ducks	Laying ducks	*P* value
Estrogen (pg/ml)	23 ± 4.4	134 ± 22.8	*< 0.001*
Progesterone (ng/ml)	0.09 ± 0.016	2.73 ± 0.478	*< 0.0001*

## Discussion

In the present study, only the left oviduct developed in the Egyptian balady duck to fill most of the left side of the abdominal cavity. The duct appeared more voluminous in laying ducks than in juvenile ones, consistent with their active role in egg production, and appeared fixed to the dorsal and ventral abdominal walls by two reflections of the mesentery, the dorsal and ventral oviductal ligaments. These observations are consistent with those noted in turkey [25]. Topographically, the duct was related to the left ovary cranially, the cloacal urodeum caudally, the left kidney dorsally, and the stomach and intestine ventrally. These relations are in line with an earlier report in the same breed of duck [11].

The oviduct of the Egyptian balady ducks was differentiated into five distinct regions: infundibulum, magnum, isthmus, uterus, and vagina, with the uterus displaying the greatest diameter in both prelaying and laying ducks. Similar oviduct subdivisions were noted in chicken [7], ostrich [26, 27], and turkey [8]. However, McLelland [28] stated the oviduct of sexually active chickens to consist of six segments: infundibulum, magnum, isthmus, tubular parts of the uterus, pouch of the uterus, and vagina.

The oviduct mucosa of the Egyptian balady ducks appeared thrown into folds separated from each other’s by furrows throughout its length. Similar mucosal organization was seen in chicken [29], Pekin duck [26], and turkey [30]. The folds were of primary, secondary, and tertiary orders, similar to those mentioned in chickens [31]. However, the latter finding was dissimilar to those reported in the oviduct of the studied duck breed, in which no tertiary folds were detected [11].

The oviduct surface epithelium consisted of non-ciliated and ciliated cells. The cilia of the latter appeared more distinctly developed in laying ducks, suggesting an enhanced activity of the ciliary machinery. Such increased ciliary activity is possibly controlled by the increased level of reproductive hormones associated with egg deposition [32].

Increased thickness of mucosal folds was a notable feature in the oviduct of Egyptian balady ducks. This finding is consistent with previous observations in chickens and ducks [33]. Since no change was detected in the thickness of the surface epithelium, the increased fold width is primarily achieved by augmented thickening of its core components. This assumption was evident in the magnum, where the proprial glands appeared condensed, highly branched, and filled most of the core of the fold.

Our transmission electron microscopic study revealed the presence of secretory granules of moderate electron density and variable sizes within the secretory cells of the proprial glands in laying ducks. The number of these granules was higher in laying ducks compared to prelaying individuals. Well-developed cisternae of the Golgi apparatus and rough endoplasmic reticulum were noticed within the proprial gland cells close to these granules, indicating their indispensable roles for synthesis, packaging, and transport of the secretory products during egg laying. Small-sized electron-dense granules were also observed within the non-ciliated secretory and ciliated cells of the lamina epithelialis. Similar appearance and distribution patterns of secretory granules were also noted in laying hens [34]. The latter finding could indicate a species-specific distribution pattern of secretory granules. Continuous release of secretory granules on the oviductal surface mucosa seems to regulate the egg passage inside the duct during its journey. The presence of furrows in between these folds enables for retention of a considerable portion of these secretions to lubricate eggs during their passage.

The present study observed intracytoplasmic vacuoles within both ciliated and non-ciliated cells of the oviduct epithelium that appeared prominent in the uterus. According to previous studies in chicken, these vacuolar spaces possibly represent disintegrated secretory granules that are reused for cellular fueling and maintaining intracellular energy supplies [35].

Interstitial Cajal-like cells, or telocytes, are specialized interstitial cells found in the stroma of various organs including those of the reproductive system [36]. They perform important functions including endocrine and neuronal signaling [37, 38]. The cells are characterized by their small bodies and elongated nuclei and long cytoplasmic processes [39, 40]. These processes extend between other cells and provide connections between different cells within the body. Telocytes are involved in multiple functions including cell proliferation, regeneration, and immune signaling [41]. Furthermore, they are crucial for protection against inflammation [42]. Telocytes were observed in chicken oviduct close to epithelial cells, smooth muscle cells, and nerve cells [43]. Similar to the mentioned study, telocytes were seen near secretory cells of the proprial glands in Egyptian ducks. Such localization may suggest a regulatory effect of these cells on the secretory activity of the duck oviduct. However, further characterization of these cells, their direct contact with different cell types in the wall of the avian oviduct, and their possible regulatory roles warrant future investigations.

Numerous studies have focused on the molecular regulation of the process of egg formation and biomineralization. However, studies that followed the immunological microenvironment of the avian oviduct are limited. T-cell-mediated immunity is crucial for protection against many avian diseases [44]. Among diseases that involve T cell activation in the oviduct are avian influenza virus [45] and duck circovirus [46]. Repeated waves of these diseases result in severe economic losses. Colonization of the oviduct wall by T cells starts by the age of five weeks, and their population is intrinsically regulated by sex hormone levels [47, 48]. Cluster of differentiation 3 (CD3) is a transmembrane protein that mediates T cells immunological activity following its binding to the short cytoplasmic tail of the T cell receptor [49]. Our immunohistochemical analysis revealed CD3 immunoreactive T cells within the oviductal mucosa of the Egyptian balady ducks, being more enriched within the lamina propria than the epithelium. The T cell distribution pattern noted by the present study could suggest a T-cell-mediated protection within the oviduct of the Egyptian balady ducks that appears accentuated by the process of egg laying.

Estrogen and progesterone are two primary steroid hormones vital to the female reproductive tract. In birds, they contribute to several important functions related to growth of the ovary and oviduct, follicle maturation, ovulation, and egg formation [50]. They are mainly synthesized by granulosa and theca cells of the ovarian follicles, especially those of preovulatory order, from which they reach the bloodstream [51]. Progesterone levels have been reported to increase in blood during the 2–7 h preceding ovulation in chicken, turkey, and duck [52–54]. Such increase has been suggested to be induced by the daily preovulatory surge of luteinizing hormone (LH) to further assist in ovum release by the selected preovulatory hierarchical follicle [55]. Higher blood estrogen levels were reported in egg-laying birds compared to juvenile ones [56]. Moreover, receptors for estrogen and progesterone are expressed by components of the oviduct wall including the cells of the surface epithelium and tubular glands [57, 58]. Experimental studies in chicken and quail proposed positive effects of exogenously administered female reproductive hormones on the growth, structural, and functional integrity of the avian reproductive tract [57, 59]. In the present study, and in view of the aforementioned information, blood samples collected around the time of ovulation in balady ducks revealed significantly higher plasma levels of estrogen and progesterone that probably occur on a daily basis to ensure proper ovulation in the ovary and sufficient raw material for egg formation by the oviduct.

This study is the first to report in-depth analysis of the structure of the oviduct in Egyptian balady ducks. The study is challenged by the number of birds, five per each technique i.e., light microscopy, electron microscopy and morphometry. However, the overall data appears coherent as the oviduct was analyzed in all birds throughout its length making the finding of each technique confirmatory for others.r

## Conclusion

In summary, the present study reported micromorphological, ultrastructural, and immunohistochemical changes in the oviduct of pre-lay and in-lay Egyptian balady ducks. These changes, which include alterations in mucosal fold thickness, distribution of mucosal T lymphocytes, ciliary function of surface epithelium, and secretory activity of the tubular gland cells, could be contributed by the increased production of estrogen and progesterone and may reflect physiological adaptations mediated by the process of egg deposition. Future studies are required to dissect molecular mechanisms governing these changes in the studied species as well as other related Aves.

## Data Availability

All datasets generated or analyzed during this study are included in the published article.
